# Cholangitis, Choledocholithiasis and a Cholecystocolonic Fistula: A Case Report on a Rare Triple Surgical Challenge

**DOI:** 10.1155/cris/1989959

**Published:** 2026-09-09

**Authors:** Kumail Jaffry, Anthony Tran, Deep Bhowmik, Amy Crowe, Niyaz Naqash

**Affiliations:** ^1^ General Surgery and Acute Surgery Unit, Monash Health, Victoria, Australia, monashhealth.org; ^2^ Department of Surgery, Monash University, Victoria, Australia, monash.edu

**Keywords:** acute cholangitis, cholecystocolonic fistula, choledocholithiasis, gallstone migration, single-stage surgery

## Abstract

Cholecystocolonic fistula (CCF) is an uncommon complication of gallstone disease and is usually diagnosed intraoperatively. Concomitant choledocholithiasis and acute cholangitis create competing priorities: urgent biliary drainage and definitive treatment of the fistula. A 78‐year‐old woman presented with 3 days of upper abdominal pain, fever, vomiting and jaundice. She was febrile and hypotensive but stabilised after intravenous fluid resuscitation without vasopressors. Laboratory and imaging findings met Tokyo Guidelines 2018 criteria for severe acute cholangitis. Computed tomography demonstrated an 11 mm impacted distal common bile duct (CBD) stone, severe biliary dilatation, pneumobilia and a large calcified gallstone in the hepatic flexure, consistent with CCF and gallstone migration. After resuscitation and correction of coagulopathy, she underwent surgery within 24 h. Laparoscopy confirmed the fistula, and intraoperative cholangiography showed multiple obstructing ductal stones. Several stones were removed transcystically; however, an impacted distal stone required conversion to open choledochotomy. Cholecystectomy, right hemicolectomy with ileocolic anastomosis, and primary CBD closure were completed during the same operation. Postoperative bilious drainage prompted further imaging and endoscopic retrograde cholangiopancreatography (ERCP). Imaging demonstrated a cystic duct stump leak, while ERCP confirmed retained choledocholithiasis. Sphincterotomy and balloon extraction achieved ductal clearance, and a plastic CBD stent was inserted. The leak subsequently resolved, and the stent was removed approximately 4 months later, with no residual choledocholithiasis identified. Histopathology excluded malignancy. This case illustrates that single‐stage biliary and colonic surgery may be feasible in a resuscitated, haemodynamically stable patient with severe cholangitis when appropriate expertise is available. It also supports a low threshold for postoperative ERCP when ductal clearance remains uncertain.

## 1. Introduction

Cholecystocolonic fistula (CCF) is the second most common cholecystoenteric fistula after cholecystoduodenal fistula [1, 2]. It accounts for ~8%–26.5% of cholecystoenteric fistulas and is identified in 0.06%–0.14% of patients undergoing cholecystectomy [1, 2]. CCF usually develops as a late consequence of chronic calculous cholecystitis, when persistent inflammation and pressure from an impacted stone cause gallbladder wall necrosis and erosion into the adjacent colon [1, 3]. It occurs predominantly in older women, and concurrent hepatobiliary disease is reported in approximately one‐quarter of patients, with gallbladder cancer reported in 2% [1].

Clinical presentation ranges from chronic diarrhoea and vague abdominal discomfort to biliary sepsis or gallstone ileus. Most CCFs are diagnosed intraoperatively, when an intended cholecystectomy may unexpectedly require colonic repair or resection in the presence of dense inflammation.

When CCF coexists with choledocholithiasis and acute cholangitis, treatment must address both infected biliary obstruction and the persistent bilioenteric fistula. Options include endoscopic or percutaneous biliary drainage followed by interval surgery, or single‐stage operative bile duct clearance and fistula management. Comparative evidence is limited, and the optimal approach remains uncertain. We report a patient treated in a single operation with laparoscopic‐to‐open bile duct exploration, cholecystectomy, and right hemicolectomy. This report was prepared in accordance with the CARE guidelines [4].

## 2. Case Presentation

A 78‐year‐old woman presented to the emergency department of a metropolitan teaching hospital with a 3‐day history of intermittent upper abdominal pain, fever, vomiting and new‐onset jaundice. She denied bowel or urinary symptoms. Her medical history included hypertension, hypercholesterolaemia and a right total hip arthroplasty. She was not taking any anticoagulant or antiplatelet agent. At baseline, she lived at home and was independent in all activities of daily living.

On presentation, she was febrile at 38.1°C and hypotensive at 90/72 mmHg. Her blood pressure normalised with intravenous crystalloid, and she did not require vasopressor support. Abdominal examination demonstrated marked right upper quadrant tenderness with involuntary guarding. Laboratory investigations showed a white cell count of 13.7 × 10^9^/L, platelet count of 95 × 10^9^/L, C‐reactive protein of 275 mg/L, alkaline phosphatase of 270 U/L, gamma‐glutamyl transferase of 313 U/L, alanine aminotransferase of 85 U/L, total bilirubin of 130 μmol/L and lactate 3.0 mmol/L. The international normalised ratio was 2.3 despite the absence of anticoagulation. This was considered likely to reflect vitamin K malabsorption in the setting of biliary obstruction.

She met the Tokyo Guidelines 2018 diagnostic criteria for acute cholangitis, with systemic inflammation, cholestasis and radiological evidence of biliary obstruction [5]. Grade III (severe) disease was assigned because of hepatic dysfunction (INR 2.3; threshold >1.5) and haematological dysfunction (platelet count 95 × 10^9^/L; threshold <100 × 10^9^/L). She had no neurological, respiratory or renal dysfunction. Her hypotension resolved with crystalloid alone, so the cardiovascular criterion was not met. She also met three Grade II criteria: age ≥75 years, white cell count ≥12 × 10^9^/L and total bilirubin ≥85 μmol/L.

**Figure 1:**
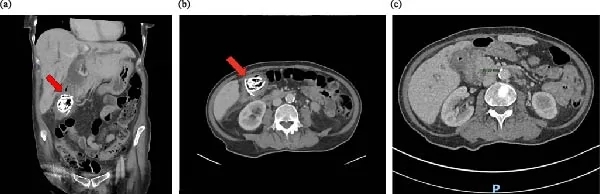
Contrast‐enhanced CT images demonstrating the cholecystocolonic fistula and associated choledocholithiasis. (a) Coronal and (b) axial images show a large peripherally calcified gallstone within the hepatic flexure, immediately adjacent to the collapsed, thick‐walled gallbladder fundus, with surrounding inflammatory change and intraluminal gas. (c) Axial image shows an ~11 mm calculus impacted in the distal common bile duct (calipers), with upstream biliary dilatation.

Contrast‐enhanced computed tomography (CT) of the abdomen and pelvis demonstrated a large calcified gallstone within the hepatic flexure adjacent to the gallbladder fundus, with colonic wall thickening, pericolic fat stranding and pneumobilia (Figure 1a,b). An 11 mm calcified stone was also impacted in the distal common bile duct (CBD), with severe intrahepatic and extrahepatic biliary dilatation and a CBD diameter of 23 mm (Figure 1c). These findings supported a diagnosis of CCF with gallstone migration, choledocholithiasis and acute cholangitis.

She was resuscitated and commenced on intravenous piperacillin‐tazobactam. Blood cultures obtained before antibiotic administration subsequently grew *Escherichia coli* and *Streptococcus anginosus* group organisms. Coagulopathy was corrected with intravenous vitamin K and two units of fresh frozen plasma. Magnetic resonance cholangiopancreatography (MRCP) was not performed because CT had already established the cause and level of biliary obstruction and demonstrated the fistula and the migrated colonic stone.

Preoperative endoscopic retrograde cholangiopancreatography (ERCP) was considered because the Tokyo Guidelines 2018 recommend urgent biliary drainage for Grade III cholangitis and favour an endoscopic approach when feasible [6]. However, ERCP would have treated only the ductal obstruction, leaving the fistula, inflamed hepatic flexure, and migrated colonic gallstone untreated. The patient stabilised promptly after fluid resuscitation and required neither vasopressors nor intensive care. The surgical unit had experience in both bile duct exploration and colorectal resection. A single‐stage operation was therefore undertaken the following morning, achieving biliary decompression within 24 h of presentation and definitive treatment of the fistula. Had her condition deteriorated or surgery proved unsafe, endoscopic or percutaneous drainage would have been pursued.

An initial laparoscopic approach was used to assess the fistula and colonic involvement, perform intraoperative cholangiography, and attempt transcystic duct clearance. Given the anticipated inflammatory distortion, the threshold for conversion was low. Access was obtained by an infraumbilical open Hasson technique, with one epigastric and two right upper quadrant ports placed under direct vision.

**Figure 2:**
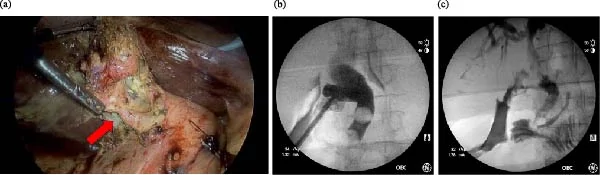
Intraoperative findings and cholangiography. (a) Laparoscopic view showing dense inflammatory adherence between the gallbladder fundus and hepatic flexure at the site of the cholecystocolonic fistula (arrow). (b) Initial intraoperative cholangiogram demonstrating a dilated cystic duct and common bile duct with multiple filling defects and no passage of contrast into the duodenum. (c) Completion intraoperative cholangiogram following bile duct exploration, interpreted as showing no residual filling defects and free passage of contrast into the duodenum.

Laparoscopy confirmed dense inflammatory adherence between the gallbladder fundus and hepatic flexure, with a CCF and a large intraluminal colonic gallstone (Figure 2a). The gallbladder was mobilised from the cystic plate, and a cholangiogram catheter was passed through the fistulous opening in the gallbladder. Intraoperative cholangiography showed a dilated cystic duct and CBD with multiple filling defects and no contrast passage into the duodenum (Figure 2b). Dissection proceeded cautiously because of the markedly distorted anatomy and obscured hepatocystic triangle [7].

Biliary decompression was prioritised. Flexible transcystic choledochoscopy and basket extraction removed several stones, but the impacted distal CBD stone could not be retrieved. The procedure was converted to an open operation through a right subcostal incision. An anterior longitudinal choledochotomy allowed extraction of the impacted calculus and further exploration of the duct.

The gallbladder was separated from the colon, and the adjacent duodenum was assessed. No macroscopic features suggested malignancy, so an oncological en bloc resection was not undertaken, although the gallbladder and the involved colonic segment were resected and submitted for histopathology. Primary repair or limited wedge resection of the colonic defect was unsuitable because the hepatic flexure was extensively inflamed and oedematous, the surrounding tissue was friable, the serosal defect was large, and a sizeable gallstone was present within the colonic lumen. A right hemicolectomy was therefore performed. The duodenum was carefully separated from the mesocolon, and intraoperative gastroscopy excluded duodenal perforation or fistulous involvement. The affected colon was resected, and an end‐to‐side stapled ileocolic anastomosis was fashioned.

Choledochoscopy through the choledochotomy retrieved additional stones from the CBD and cystic duct. Completion cholangiography was interpreted as showing no residual filling defects and free contrast passage into the duodenum (Figure 2c). The choledochotomy was closed primarily. The cystic duct stump was closed and reinforced, and a drain was placed in Morison’s pouch adjacent to the cystic duct stump and choledochotomy.

Bilious drain output persisted postoperatively. A hepatobiliary iminodiacetic acid (HIDA) scan on postoperative day 4 and CT cholangiography on postoperative day 7 were consistent with a cystic duct stump leak, a residual gallbladder fossa collection, and a possible retained distal CBD calculus. ERCP on postoperative day 8 confirmed retained choledocholithiasis. Biliary sphincterotomy and balloon extraction achieved complete ductal clearance, and a plastic stent was placed in the CBD. The bile leak subsequently resolved. The residual collection was treated with intravenous antibiotics and did not require percutaneous drainage or reoperation. Approximately 4 months later, repeat ERCP demonstrated a markedly dilated main bile duct. The stent was removed, and balloon sweeping identified no residual stones.

**Figure 3:**
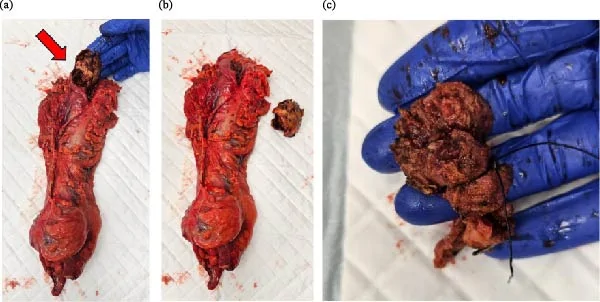
Gross pathological specimens. (a) Resected right hemicolectomy specimen showing a large calcified gallstone within the hepatic flexure (arrow). (b) The same specimen after removal of the gallstone, demonstrating marked colonic wall thickening and a 20 mm serosal defect at the site of the cholecystocolonic fistula. (c) Fragmented gallbladder measuring 50 × 30 × 20 mm, with multiple full‐thickness defects measuring 5–15 mm, consistent with fistulous communication.

Gross examination of the right hemicolectomy specimen showed a large calcified gallstone within the hepatic flexure, marked colonic wall thickening, and a 20 mm serosal defect at the site of the fistulous communication (Figure 3a,b). The resected gallbladder measured 50 × 30 × 20 mm, was fragmented, and contained multiple full‐thickness defects consistent with fistulous communication (Figure 3c). Microscopically, the gallbladder showed acute‐on‐chronic cholecystitis. The colonic specimen contained a fibrin‐lined fistula tract with inflamed granulation tissue, surrounding stromal fibrosis, adjacent mucosal ulceration and inflammatory pseudopolyps. Neither specimen showed dysplasia nor malignancy, and the resection margins were clear.

After the bile leak resolved, no further surgical complications occurred. She was discharged 8 weeks after surgery to a transitional care facility. The prolonged admission was primarily related to discharge planning and bed availability rather than ongoing surgical morbidity. Approximately 4 months after the initial ERCP, the biliary stent was removed at repeat ERCP, with no residual choledocholithiasis identified on balloon sweeping. Further follow‐up occurred outside our health service, and no subsequent readmission to our health service was recorded within 10 months of discharge.

## 3. Discussion

This case was notable for the preoperative diagnosis of CCF and concurrent choledocholithiasis causing Grade III acute cholangitis despite rapid haemodynamic stabilisation. These factors informed the decision to pursue definitive treatment in a single operation.

Although most CCFs are diagnosed intraoperatively [1], CT established the diagnosis before surgery in this patient. Recognised radiological features include pneumobilia, an ectopic gallstone, focal bowel‐wall thickening, and pericholecystic inflammation [3, 8]. The combination of a calcified gallstone in the hepatic flexure, pneumobilia, and a collapsed, thick‐walled gallbladder allowed the fistula to be anticipated. This allowed the consent process to include possible colonic resection and conversion to open surgery, prompted assessment for duodenal involvement, and ensured that biliary and colorectal expertise was available. Pneumobilia, chronic diarrhoea and vitamin K malabsorption have been described as a characteristic triad of CCF [9], although our patient did not report diarrhoea.

For Grade III acute cholangitis, the Tokyo Guidelines 2018 recommend urgent biliary drainage and favour an endoscopic approach where available [6]. A staged strategy of ERCP with sphincterotomy and stone extraction, followed by interval fistula repair after resolution of sepsis and inflammation, would have provided less invasive initial decompression and allowed further physiological optimisation. It may also have reduced the likelihood of retained ductal stones and postoperative bile leakage. However, ERCP would not have treated the fistula, diseased colon, or migrated gallstone, and it would have required at least two procedures. ERCP also carries risks of pancreatitis, bleeding and perforation [10]. In this case, the patient’s prompt response to resuscitation, improving physiology, and the availability of appropriate biliary and colorectal expertise supported a single‐stage approach. The choice between staged and single‐stage treatment should be individualised according to the patient’s physiology, anatomy, local endoscopic availability, and operative capability.

Laparoscopic bile duct exploration is an established alternative to preoperative ERCP in selected patients and can achieve high ductal clearance rates [11]. In this case, laparoscopy allowed direct assessment of the fistula and an initial transcystic attempt at duct clearance. Conversion to open surgery was required when the impacted distal stone could not be retrieved and the anatomy remained distorted by inflammation. Despite choledochoscopy and completion cholangiography that appeared satisfactory, a residual stone or fragment was missed. Marked duct dilatation can reduce the conspicuity of small filling defects. Difficult extraction may also produce fragments or sludge, while pneumobilia and inflammatory debris can impair both cholangiographic interpretation and choledochoscopic visualisation.

Primary closure of the CBD is supported in selected patients and avoids complications associated with T‐tube drainage, including dislodgement and biliary peritonitis after removal [12–14]. A T‐tube could have provided decompression and postoperative access to the duct in this case; however, it is not routinely required after apparently complete clearance and carries its own morbidity. When extraction is difficult or confidence in completion imaging is limited, planned postoperative ERCP should be considered.

The retained stone may have increased intraductal pressure and contributed to the cystic duct stump leak. ERCP treated both problems by removing the stone and reducing the transpapillary pressure gradient, allowing the leak to seal [15]. A postoperative bile leak or concern for residual obstruction should therefore prompt early endoscopic assessment.

The extent of colonic resection should reflect the local pathology. A small fistula in a healthy colon may be managed by primary repair or limited resection, whereas extensive inflammation, friable tissue, a large defect, an intraluminal gallstone, or concern for malignancy favours formal resection [16, 17]. In this patient, the large defect, severe inflammation and intraluminal gallstone supported right hemicolectomy. Histopathological examination was important because gallbladder malignancy has been reported in association with CCF, and chronic gallstone disease is a risk factor for biliary tract cancer [1, 18].

## 4. Conclusion

CCF complicated by choledocholithiasis and acute cholangitis requires urgent biliary decompression as well as definitive treatment of the fistula. In a resuscitated, haemodynamically stable patient, single‐stage biliary and colonic surgery may be appropriate when the required expertise is available. Staged endoscopic drainage followed by interval surgery remains a valid alternative. Difficult stone extraction or uncertain completion imaging should prompt a low threshold for postoperative ERCP.

## Author Contributions

**Kumail Jaffry:** writing – original draft (lead), resources (equal), writing – review and editing (equal). **Anthony Tran:** writing – review and editing (supporting). **Deep Bhowmik:** writing – review and editing (supporting). **Amy Crowe:** writing – review and editing (supporting). **Niyaz Naqash:** conceptualisation (lead), supervision (equal), writing – review and editing (equal).

## Funding

The authors received no specific funding for this work.

## Consent

Written informed consent was obtained from the patient for the publication of this case report and accompanying images. A copy of the written consent is available for review on request.

## Conflicts of Interest

The authors declare no conflicts of interest.

## Data Availability

Data sharing is not applicable to this article because no new datasets were generated or analysed. The relevant de‐identified clinical information is contained within the article.
